# Metastatic meningioma: a case series and systematic review

**DOI:** 10.1007/s00701-023-05687-3

**Published:** 2023-07-26

**Authors:** Vratko Himič, Richard J. Burman, Daniel M. Fountain, Monika Hofer, Laurent J. Livermore, D. Sanjeeva Jeyaretna

**Affiliations:** 1 Department of Neurosurgery, John Radcliffe Hospital, University of Oxford, Oxford, UK; 2 Nuffield Department of Clinical Neurosciences, John Radcliffe Hospital, University of Oxford, Oxford, UK; 3 Weatherall Institute of Molecular Medicine, University of Oxford, Oxford, UK; 4 Department of Neuropathology, John Radcliffe Hospital, University of Oxford, Oxford, UK

**Keywords:** Metastatic, Meningioma, Recurrent, Palliative, Oncology, Screening, (Epi)genomics

## Abstract

**Background:**

Meningiomas are the most common primary intracranial tumor. While the majority of meningiomas are benign, rarely they can metastasize extracranially. There is a need for a more comprehensive review of these patients to improve our understanding of this rare phenomenon and its prevalence globally. Here we describe our institution’s experience of patients presenting with metastatic meningiomas. We further perform a systematic review of the existing literature to explore common features of this rare manifestation of meningioma and review the efficacy of current treatments.

**Methods:**

We performed a retrospective clinical review of all adult patients with metastatic meningioma managed at our institution over the past 20 years, identifying 6 patients. We then performed a systematic review of cases of metastatic meningioma in the literature ranging from the years 1886 to 2022. A descriptive analysis was then conducted on the available data from 1979 onward, focusing on the grade and location of the primary tumor as well as the latency period to, and location of, the metastasis.

**Results:**

In total, we analyzed 155 cases. Fifty-four percent of patients initially presented with a primary meningioma located in the convexity. The most common site of metastasis was the lung. Risk factors associated with a shorter time to metastasis were male sex and a high initial grade of the tumor. Regarding treatment, the addition of chemotherapy was the most common adjunct to the standard management of surgery and radiotherapy. Despite an exhaustive review we were unable to identify effective treatments. The majority of published cases came from centers situated in high-income countries (84%) while only 16% came from lower- and middle-income countries.

**Conclusions:**

Metastatic meningiomas pose a pertinent, and likely underestimated, clinical challenge within modern neurosurgery. To optimize management, timely identification of these patients is important. More research is needed to explore the mechanisms underlying these tumors to better guide the development of effective screening and management protocols. However, screening of each meningioma patient is not feasible, and at the heart of this challenge is the inability to control the primary disease. Ultimately, a consensus is needed as to how to correctly screen for and manage these patients; genomic and epigenomic approaches could hold the answer to finding druggable targets.

**Supplementary Information:**

The online version contains supplementary material available at 10.1007/s00701-023-05687-3.

## Introduction

Meningiomas are the most common primary neoplasm of the central nervous system [42]. Traditionally thought to derive from arachnoid cap cells, the more common meningiomas are more likely to derive from the arachnoid barrier cells or dural border cells [21]. While most of these tumors have a benign course and a relatively good prognosis, a small subset, approximately 1–3%, become malignant and invade local intracranial structures [13, 18]. Furthermore, in less than 1% of patients these tumors metastasize to distant extracranial locations [41]. Extracranial manifestation of malignant meningiomas was first reported in 1886 [36], and since then there have been reports describing varied presentations of this rare phenomenon.

Patients with metastatic meningioma (MM) often have a poor prognosis. Due to their rarity, there is a dearth of effective screening and management protocols for these patients [16]. Improved and more targeted detection of these cases is needed, as many patients can go several decades before the metastatic disease is found, often on incidental imaging, by which point the care is palliative [10]. Crucially, no effective treatments exist for these patients.

To add to the current clinical knowledge of metastatic meningioma, we present a case series of six patients from a single center in the UK. We describe the presentation of these patients as well as a reflection on the management strategy adopted for each case. We then systematically review the existing literature to summarize the findings of all published cases of this rare metastatic tumor. We then explore the relationships between the primary tumor and the metastatic lesions in a descriptive analysis. In an attempt to expand the global relevance of this topic, we stratify the cases across countries and economic classifications.

## Methods

### Case series

Between January 2002 and January 2022, six patients presented to our institution with metastatic meningioma. Clinical information was collected from medical records (Table 1) [30]. Only adult patients (≥ 18 years) were included. Extracranial metastases were confirmed on histopathology performed by an experienced neuropathologist. We did not include metastases within the cranium, “drop” metastases to the spinal cord, or those due to local or iatrogenic spread (i.e., spread to the tissues overlying the incision site from the surgery). None of our patients had a diagnosis of genetically inherited syndromes such as neurofibromatosis type 2 (NF2). The use of clinical information was approved by the Oxford University Hospitals Foundation Trust Clinical Audit Committee.

**Table 1:** Case series of metastatic meningioma cases from a single center

Age and sex	Histology	CNS WHO grade	TTM (years)	Primary location	Metastasis location	Further details	Initial management	Current management
59 F	Anaplastic	3	7	Right occipital	Liver and peritoneum	Increased signal in colon, breast, thyroid, and T7	Resections and radiotherapy	Radiotherapy and surveillance MRI, 500 mg of dexamethasone
70 F	Atypical	2	14	Right frontal	Parotid	Excised with right superficial parotidectomy	Resections and radiotherapy	Died 20 years after first surgery
63 M	Anaplastic	3	1	Parasagittal	Lung	Single nodule	Resections and radiotherapy	No further treatment options and referred to palliative care
47 M	Anaplastic	3	2	Left convexity	Bone (extracranial)	C7 with C7/8 compression, T2, T5, T9, right sacrum, acetabulum, femur, sternum, 5th rib	Resections and radiotherapy	Spinal radiotherapy
46 M	Atypical	2	3	Middle cranial fossa	Liver, lung, kidney, long bone, spine	T5 vertebral extending into canal	Resections and radiotherapy	Died 3 years and 2 months after first surgery
59 M *	Atypical	2	2	Falx/SSS junction	Skin, pleura, liver, bone	Right iliac bone	Resections and radiotherapy	Died 3 years and 1 month after first surgery

Six patients from the records of the Oxford Neurosurgery Department at the John Radcliffe Hospital in Oxford

*This case has already been published by McCarthy et al. (2016) [30]; we have not included a description of the case but use it in our analysis

*TTM*, time to metastasis

### Systematic review

VH and RJB performed a search on the MEDLINE database with the following MeSH terms: “metastatic meningioma,” “meningioma metastasis,” and “meningioma metastases.” Forward and backward citation searching was used to capture studies that were not found in our original search. A full list of articles found through this search was pooled together. A first round of selection was then completed independently by VH and RJB by reviewing the title and abstract of the listed studies to ensure they capture the key terms listed above and a full text was accessible. Next, a full-text review was independently reviewed and assessed for inclusion or exclusion. If there was discordance in the decision, the study was reviewed by VH and RJB together with DSJ, and a consensus was reached. Data extraction was completed using a common data template that was constantly reviewed. Studies were excluded if they pertained to the following: tumor-to-tumor metastasis within the primary meningioma, ectopic primaries, “drop” metastases to the spinal cord, or iatrogenic “metastases” (i.e., spread directly to the tissues overlying the incision site from the surgery). Only studies on adult patients were included. Studies were also excluded if they described patients who were found to have older (now-obsolete) classifications on histology. This was of particular importance in earlier descriptions of these patients where the distinction between meningiomas and hemangiopericytomas as separate tumor diagnoses was not yet well established, and descriptors like angioblastic, fibroblastic, and endotheliomatous (in the absence of a CNS WHO grade) cannot be interpreted from the older literature. Only full texts were included; if one could not be obtained through the University of Oxford Library Services or by subsequent request from international libraries and repositories, it was excluded. Papers in languages other than English were translated and, where possible, confirmed with a native speaker. We only included studies published after the release of the publication of the first CNS WHO tumor grading system in 1979. The search was conducted using the PRISMA guidelines [33]. A summary of the studies included is provided in Supp. Table 1.

### Descriptive analysis

To summarize features reported across studies, we performed an analysis specifically focusing on the following features: patient age, sex, location, and CNS WHO grade of the primary intracranial meningioma, time to first metastatic lesion (TTM), and the MM location. We categorized the location of the tumors into those at the skull base (e.g., anterior, middle, and posterior fossa), convexity (e.g., frontal, parietal), falx/tentorium, and others (e.g., intraventricular).

### Statistics

Normally distributed data were reported as mean with standard error of the mean (SEM), while non-normally distributed data were reported as median with interquartile range (IQR). Parametric and non-parametric statistical tests were used to assess the difference between variables. Significance was defined as *p* = 0.05 to test multiple comparisons (with Dunn’s correction). All plots and statistical analyses were generated using custom-made Python code (available upon request).

## Results

### Case series from local institution

#### Case 1

A 51-year-old female presenting with left homonymous hemianopia was operated on for a primary mass arising from the tentorium abutting the occipital lobe. This was confirmed as an anaplastic (CNS WHO grade 3) meningioma. Five years later, she was found to have intracranial extension of the meningioma to a bilateral distribution, including parafalcine, parasagittal, tentorial, and the right convexity. She developed mild left-sided weakness and experienced seizures on several occasions affecting her left side. An FDG-PET scan 7 years after her initial presentation in 2014 showed metastases in the liver and peritoneum. Further imaging since has shown increased signal in the breast, T7 vertebra, and thyroid enlargement, although these could be related to a different colonic mass. The patient has received radiotherapy and is having surveillance MRI scans. Her symptoms are managed with 500 mg of dexamethasone. Progression was noted on her most recent scan and whole genome sequencing is being performed to determine druggable targets.

#### Case 2

This 50-year-old woman underwent an operation for a right frontal meningioma of atypical (CNS WHO grade 2) histopathology. She underwent two more resections over the course of the next decade and, 12 years after the diagnosis, completed radical radiotherapy (54 Gy in 30 fractions) for local recurrence and subgaleal extension. Fourteen years after the first operation, she was found to have a right parotid metastasis which was resected. The aggressive nature of the intracranial tumor meant that she lost the functional use of her arm and leg on her left side. She died 20 years after her first surgery.

#### Case 3

**Fig. 1:**
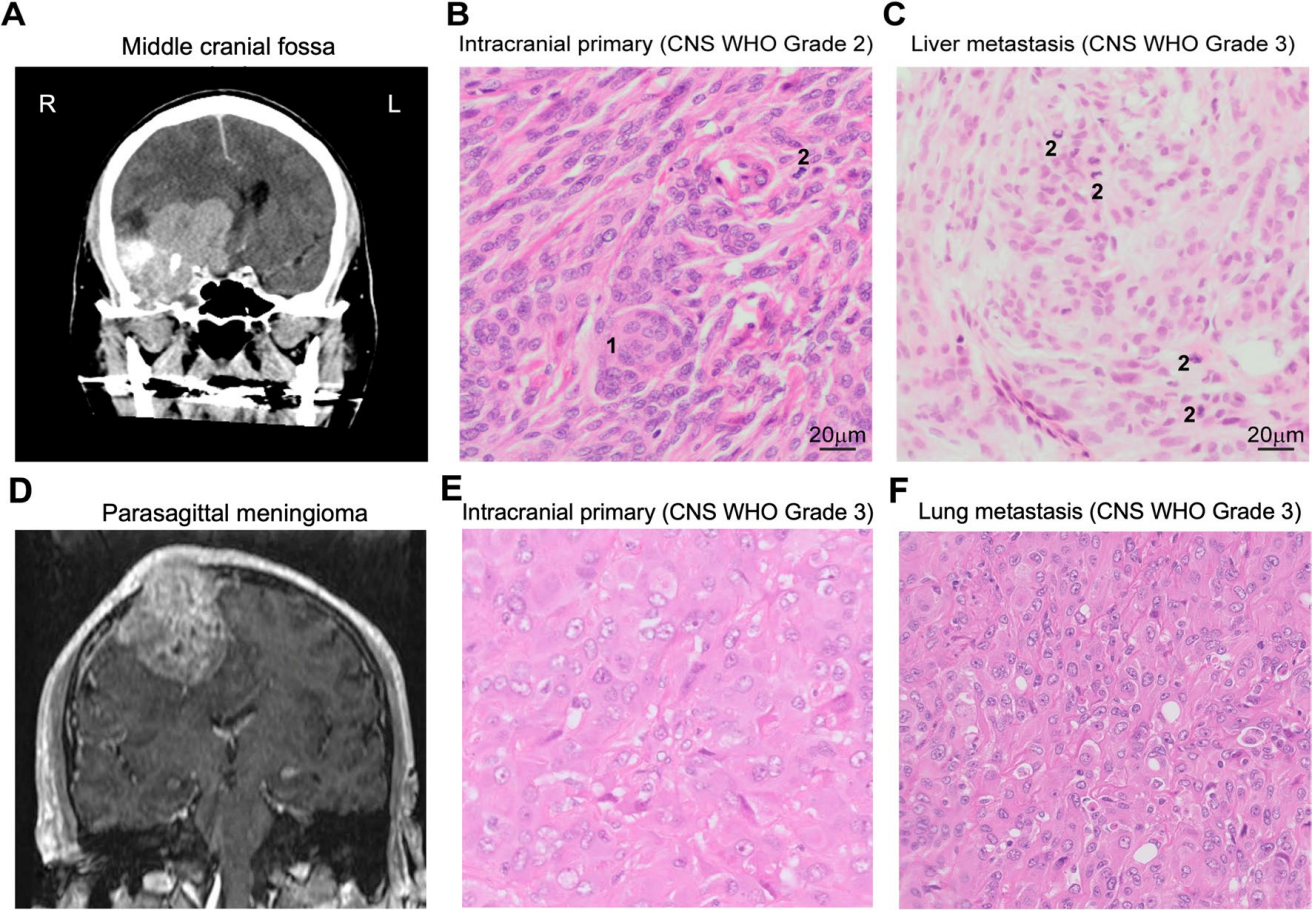
Two example cases. **A–C** Extracranial metastasis to the liver.** A** A CT scan with gadolinium showing an intracranial primary meningioma before surgery at the right middle cranial fossa (case 5). **B** Photomicrograph of hematoxylin and eosin (HE)-stained section of temporal resection showing characteristic whorl (1) and a single mitotic figure (2). Mitotic activity was present between four and 20 per 10 high power fields. The primary tumor was diagnosed as an atypical intracranial meningioma and classified as CNS WHO grade 2. **C** Photomicrograph of HE-stained section of liver biopsy showing four mitotic figure (2). Mitotic activity is present in more than 20 per 10 high power fields. Tumor was diagnosed as a metastatic anaplastic meningioma and classified as CNS WHO grade 3. **D–F** Extracranial metastasis to the lung.** D** A T1-weighted MRI scan showing a parasagittal primary meningioma with demonstration of the patient’s presenting complaint; a skin “lump” due to the extension of the neoplasm (case 3). **E** and **F** Both the primary (**E**) and lung metastasis (**F**) show an anaplastic meningioma, CNS WHO grade 3 (mitotic activity > 20 per 10 high power fields). Sheet-like architecture and some rhabdoid morphology can be seen

A 61-year-old male presented with a 1-month history of a lump on the top of the head with 5 weeks of generalized headache that was non-radiating. This lump was initially fluctuant but hardened over time, leading to a diagnosis of a sebaceous cyst. As penicillin did not reduce the size of the lump, their primary care physician referred the patient for further investigations. The patient also developed uncoordinated movements with frequent falls. He was persistently lethargic with significant weight loss. Imaging revealed a mass in the right parasagittal region. He underwent resection of the primary tumor which was an anaplastic meningioma (CNS WHO grade 3). One year later he was found to have a single lung metastasis alongside recurrence at the primary lesion site, he underwent a lobectomy for the lung mass and re-operation for the cranial recurrence (Fig. 1). Follow-up PET imaging showed enhancement in the hilar region, strongly suggestive of further metastatic spread. He subsequently underwent radiotherapy and further surgery to control seizures. The patient has since been transferred to palliative care.

#### Case 4

This 44-year-old man presented with a painless, firm, 5-cm skin lump on the scalp that grew significantly over a 2-month period. MRI showed that this mass was durally based, eroding bone, and extending into the temporalis muscle with no cerebral edema. CT of the chest abdomen and pelvis (CT CAP) showed no primary lesion. The tumor was located at the left convexity and had a significant extra-osseus component. While the tumor was initially thought more likely to be an osteosarcoma, histopathological analysis revealed an anaplastic meningioma (CNS WHO grade 3). The tumor recurred, including in the orbital apex, leaving the patient with rapid visual loss and exophthalmos. Two years after his initial surgery, whole body FDG-PET showed metastases to the vertebrae, sacrum, femur, and sternum, showing a particular predilection for bone, alongside recurrence in the posterior fossa. After repeated resections of the skull base lesion, the patient is receiving radiotherapy for spinal metastases.

#### Case 5

A 46-year-old man presented with a 6-month history of decreased visual acuity in the right eye. Imaging revealed a lesion in the right middle cranial fossa that involved the bone with orbital and infratemporal fossa extension. Following resection this was shown to be an atypical (CNS WHO grade 2) meningioma. Eight weeks after the surgery the patient developed a CSF leak and subsequent wound infection. This was treated with a lumbar drain and broad-spectrum antibiotics. Post-operative imaging a year later revealed a temporal lobe collection with osteomyelitis of the sphenoid wing. The drained pus suggested infection with Propionibacterium, again treated with antibiotics. For two more years the patient was treated with focal radiotherapy for tumor recurrence. Three years since the initial presentation, the gentleman developed right facial weakness and an ipsilateral clival lesion was detected on imaging. Following this, a whole-body CT with contrast showed extensive metastatic disease, including to the vertebrae, long bones, liver, kidney, and lung. The patient received urgent radiotherapy to combat the radiological cord compression. A biopsy of a hepatic lesion suggested a metastatic anaplastic (CNS WHO grade 3) meningioma. A course of palliative chemotherapy was scheduled, but unfortunately the patient died 3 years and 2 months after their initial surgery. Imaging and histology for this patient are shown in Fig. 1.

### Systematic review and analysis

**Fig. 2:**
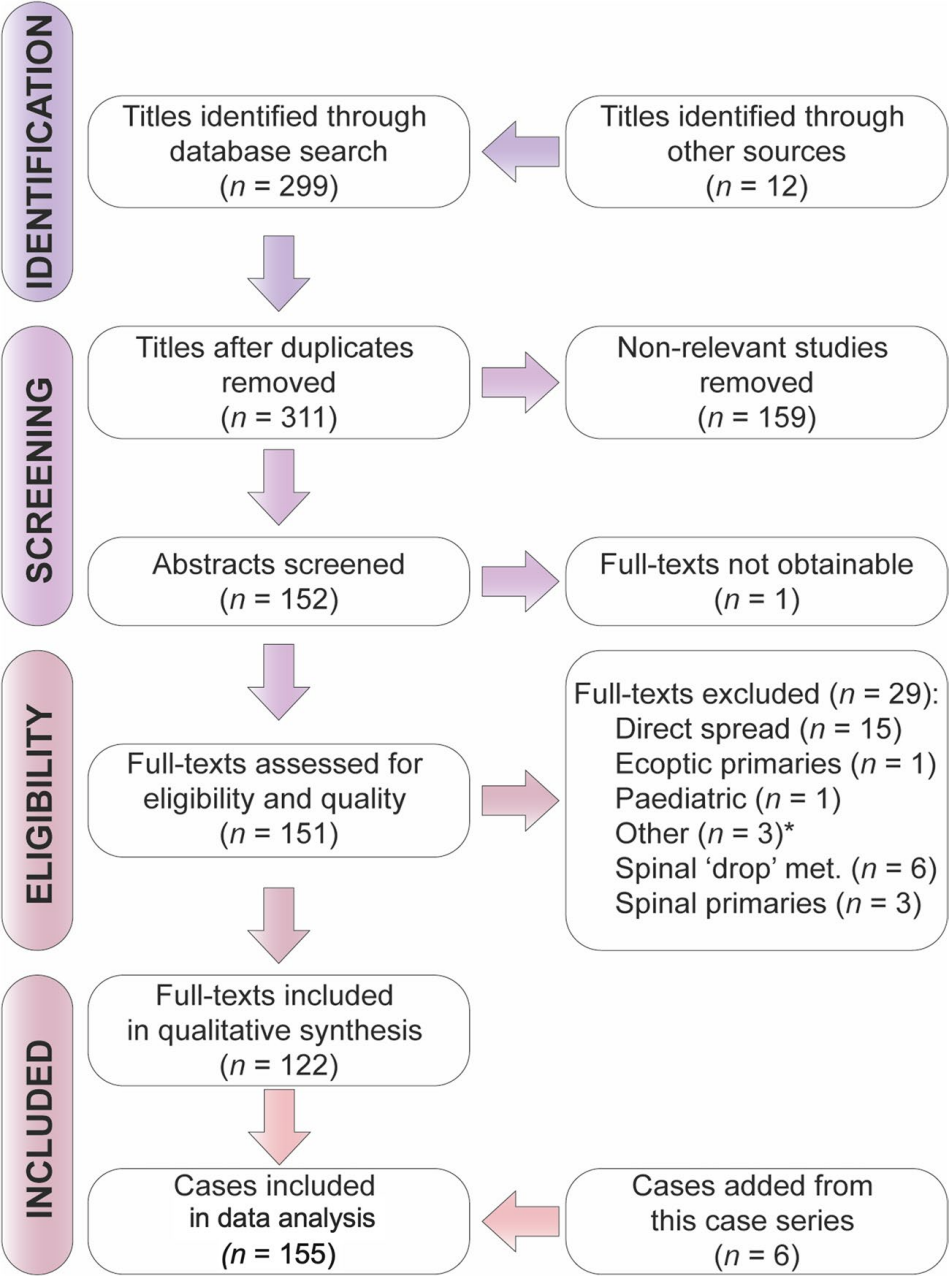
PRISMA flowchart. “Other” (*) includes patients who were initially classified as having primary intracranial meningiomas which were later found to be hemangiopericytomas on histology

Through our literature search we identified 299 titles which described studies on MM (Fig. 2). After exclusions we were left with 122 full-text manuscripts to review from which we collected clinical data from 149 cases. We then combined this data with our local case series giving a total of 155 cases.

**Fig. 3:**
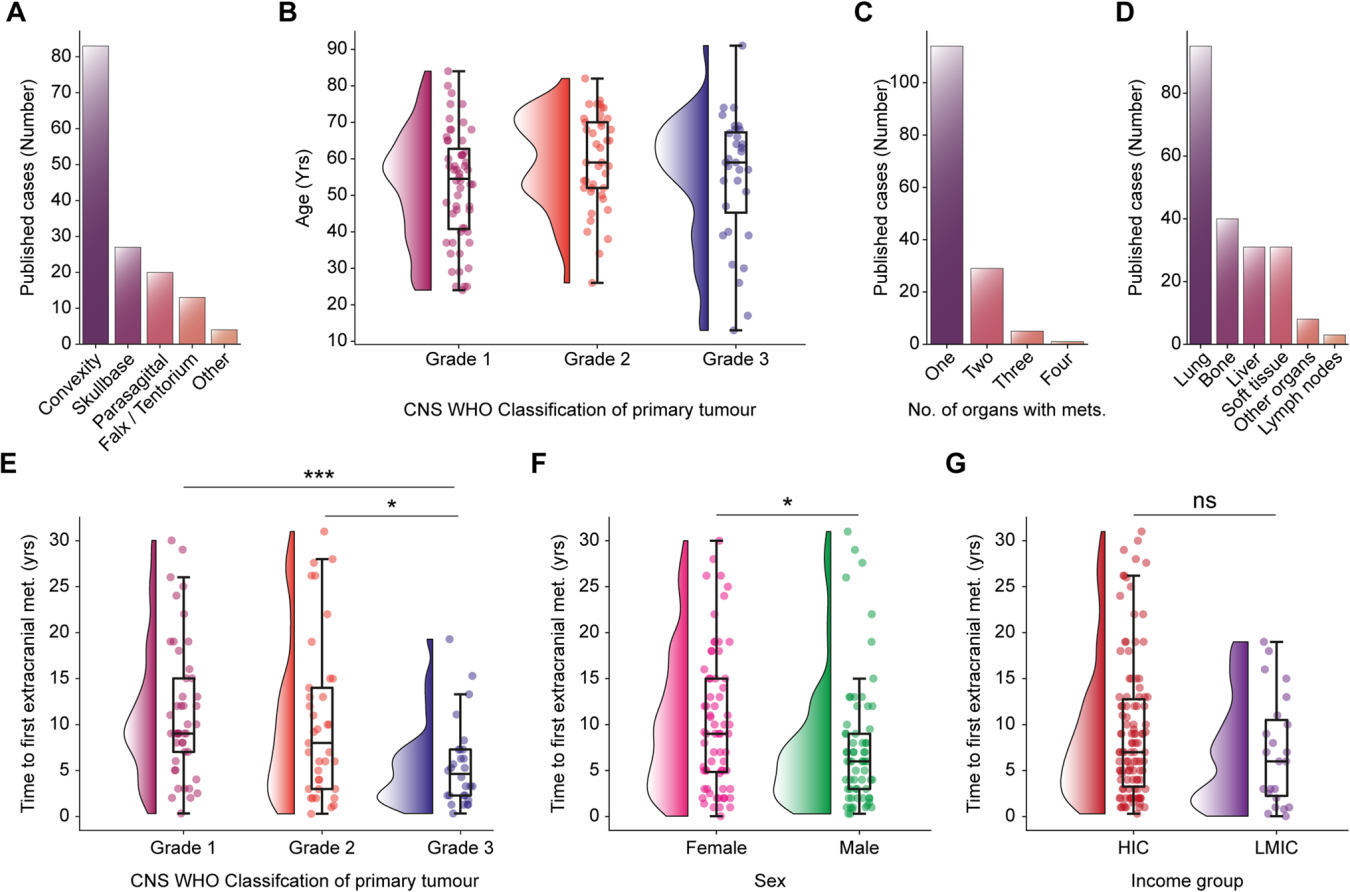
Age distribution, grade, and location of primary intracranial tumor. **A** Location of primary intracranial meningioma. **B** No difference in age of patients presenting with different CNS WHO grades of primary intracranial meningioma (grade 1: mean: 54.5 ± SEM 2.1 years; grade 2: mean: 59.59 ± SEM: 2.0 years; grade 3: mean 55.1 ± SEM 3.1 years; *p* = 0.57, *one-way ANOVA test*). **C** Bar plot of number of cases which presented with a single organ or multiple organs containing extracranial metastases. **D** The number of cases in which metastases were found in specific organs. **E** The CNS WHO grade of the primary intracranial meningioma significantly affects the time to first presentation with an extracranial metastasis (*p* = 0.0003, *Kruskal–Wallis test*). Patients with CNS WHO grade 3 primary intracranial meningiomas present with extracranial metastases sooner compared to those with CNS WHO grade 1 (grade 3: *median* 4.4 *IQR* 5.0 years vs grade 1: *median* 9 *IQR* 8.5 years, *p* = 0.0002, *Dunn’s multiple comparisons test*) or CNS WHO grade 2 (grade 2: *median* 8 *IQR* 11.5 years, *p* = 0.02, *Dunn’s multiple comparisons test*). There was no difference between grade 1 and grade 2 tumors (*p* = 0.76, *Dunn’s multiple comparisons test*). **F** Differences in sex and time to first extracranial metastatic lesion (female: *median* 9.0 *IQR* 10.3 years vs male: *median* 6.0 *IQR* 8.75 years, *p* = 0.0003, *Mann–Whitney test*). **G** Differences time to first extracranial metastatic lesion and the income group of the country in which the patient was treated (high-come country, HIC: *median* 7.0 *IQR* 10.0 years vs low-middle-income country, LMIC: *median* 6 *IQR* 8 years, *p* = 0.16 *Mann–Whitney test*)

In the full cohort, 52% of patients were female (sex not reported in 1 patient) with a median age of 56 years (IQR 25 years). In most cases (54%), the primary intracranial meningioma was located at the convexity (Fig. 3A). Thirty-six percent of patients presented with a grade 1 intracranial primary (grade 2: 26%, grade 3: 21%, not reported: 17%). There was no difference in the age of patients presenting with different CNS WHO grades of intracranial meningiomas (grade 1: mean: 54.5 ± SEM 2.1 years; grade 2: mean: 59.59 ± SEM: 2.0 years; grade 3: mean 55.1 ± SEM 3.1 years; *p* = 0.57, *one-way ANOVA test*) (Fig. 3B). We also summarize the reported histological descriptions by subtype (Supplementary Table 2).

Most patients (74%) presented with metastases affecting a single organ, with the lung being the most commonly affected (61%, Fig. 3D). Other common sites included extracranial bone (26%), liver (19%), and soft tissue (19%). We found that the CNS WHO grade of the primary tumor significantly affects the latency when patients present with extracranial metastases (Fig. 3E). Patients with grade 3 primary tumors presented with extracranial metastases sooner than those with grade 1 tumors (grade 3: median 4.4, IQR 5.0 years vs grade 1: median 9, IQR 8.5 years, *p* = 0.0002, *Dunn’s multiple comparisons test*) or grade 2 (grade 2: *median* 8 *IQR* 11.5 years, *p* = 0.02, *Dunn’s multiple comparisons test*). We found a significant sex bias in the TTM with male patients presenting sooner than female patients (Fig. 3F). Furthermore, there was no difference between the time to presentation and the socioeconomic status of the country in which the patient was based in Fig. 3G. There was no correlation between the age of patients and the TTM (Supp. Figure 1).

**Fig. 4:**
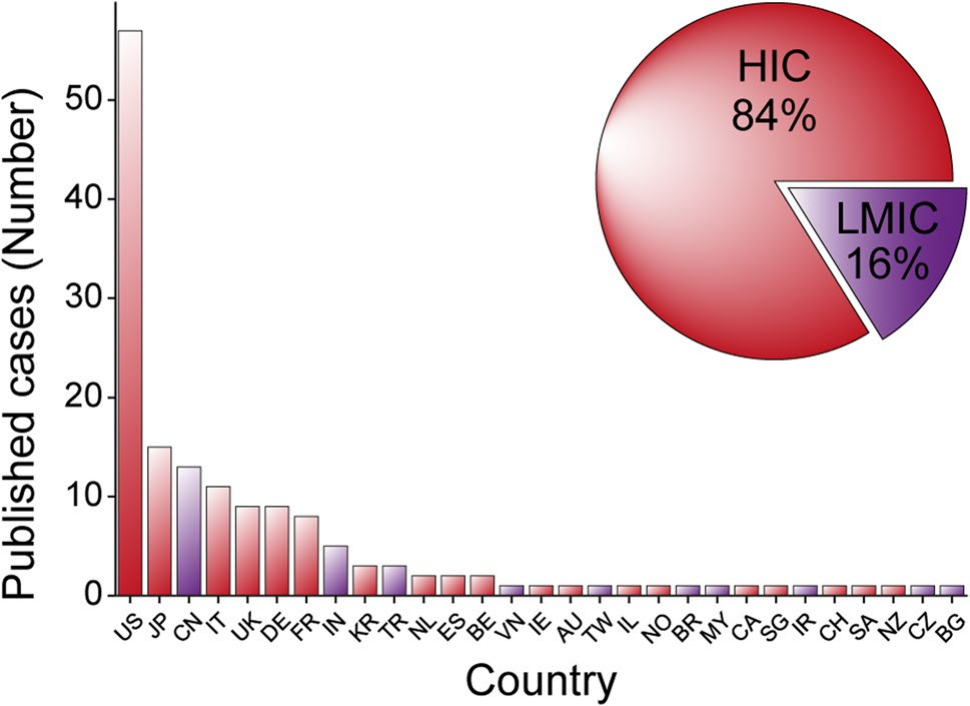
Geographic and economic distribution of published cases. The bar plot stratifies the number of cases published from each country. The pie plot (inset) separates cases as per the income group of the country from which the institution they came from is situated. Income groups where defined as per data from the World Bank based on gross national income (GNI) per capita countries were either classified as high-income (HIC) or low-middle-income (LMIC) which includes low-income, lower-middle income, and upper-middle-income

The majority of published cases came from centers situated in high-income countries (HIC, 84%) while only 16% came from lower- and middle-income countries (LMIC) (Fig. 4) [43]. The USA has published the greatest number of the cases on this topic (57) with the closest follower, Japan (JP), publishing 15.

Many of the patients we reviewed were treated with novel management plans owing to lack of established protocols. In addition to standard surgical resection and radiotherapy, we found that 34 cases had received an additional treatment, with classical chemotherapeutic agents like hydroxyurea being the most commonly used (Table 2). We were unable to identify a consistent therapy with a clear survival benefit.

**Table 2:** Treatments trialed for metastatic meningiomas

Novel therapy trialed	Number of times trialed
Tyrosine kinase inhibitors	6
Sunitinib	3
Valatinib	2
Apatinib (targets VEGFR2)	1
Synthetic somatostatin	8
Octreotide	8
Immunotherapy	6
Bevacizumab (anti-angiogenic)	3
Nivolumab (anti-PD-1)	2
Pembrolizumab (anti-PD-1)	1
Classical chemotherapy	34
Hydroxyurea	14
Temozolomide	5^a^
Carboplatin	3^b^
Doxorubicin	3^c^
Paclitaxel	2^d^
Vincristine-cyclophosphamide-doxorubicin	2
Ifosfamide	2^e^
Etoposide	2
Cisplatin	1^f^
Others	2
Thalidomide	1
Interferon therapy	1
Novel radiotherapeutic approaches	3
Peptide receptor radionucleotide therapy	2
Boron neutron capture therapy (BNCT)	1

A summary of novel and innovative pharmacological therapies as well as novel radiotherapeutic approaches alongside more traditional chemotherapies. All patients received surgery and standard radiotherapy

^a^Treated once with hydroxyurea/temozolomide; ^b^treated once with paclitaxel and once with etoposide; ^c^ treated once with ifosfamide; ^d^treated once with paclitaxel; ^e^treated once with doxorubicin; ^f^ treated once with carboplatin and once with cisplatin

## Discussion

Since Power’s report of the first supposed recorded case of MM in 1886 [36], the incidence of reports has been relatively low. To date there have been a few attempts to summarize the findings of the literature on this topic, initially in 1963 and most recently in 2013 [15, 40]. However, these reviews only covered a subset of the available literature. As such, there is a need for a more recent and unified understanding of the relevant descriptive features on these tumors.

The prevalence of distant metastases among all patients with meningioma is low with the most recent reports being 0.18% [41]. Indeed, these cases represent a relatively rare occurrence in what is otherwise a benign neoplasm that has, in most cases, favorable surgical outcomes [4]. Nevertheless, the TTM for many of the cases we reviewed is in the order of decades, representing opportunity for diagnosis and a window for intervention (particularly given the changing surgical paradigm of more aggressive and targeted approaches). By way of example, in the case reported by Dincer and colleagues in 2020, a 41-year-old patient was found to have pleural metastases from an intracranial meningioma. A retrospective search from old CT imaging found that it was present 13 years earlier [12]. Another layer of complexity is that only 51% of patients with systemic metastases were symptomatic at the time of detection of the metastases [40].

We found that the convexity was the most common primary location. This finding was also the case in other studies where convexity tumors were shown to be of highest prevalence [39, 45]. Meningiomas that are not located at the skull base have been shown to have double the risk of CNS WHO grade 2 and 3 pathology relative to skull base tumors [22]. In addition, larger meningiomas are more likely to be CNS WHO grade 2 [29]. A retrospective study of over 1600 cases found that skull base meningiomas had a relatively low malignant potential [8].

Our finding that the lung (61%), bone (26%), and liver (19%) were the most common sites of extracranial metastasis, in that order, was similar to the findings in a systematic review by Surov et al. in 2013 [40] with a frequency of 37.2%, 16.5%, and 9.2%, respectively. While the causes of extracranial metastases from meningioma are largely unknown, one explanation could be through iatrogenic seeding. The disruption of the tumor during resection of the primary lesion could lead to metastatic spread via the lymphatics and venous sinuses to further tissues; much in the same way as CSF is hypothesized to be the cause of “drop” intraspinal metastases [5, 40]. Knowing the most frequent locations of metastasis will be pertinent in the development of any future targeted screening pathways.

After first biopsy, we found that 47% of the tumors were CNS WHO grade 1, 31% were grade 2, and 22% were grade 3. Surov and colleagues reported that 56.2% of metastatic cases originated from CNS WHO grade 1 or 2 tumors [40]. We elected to designate the grade of the tumor as the one that was reported at primary biopsy. In several cases, a progression from CNS WHO grade 1 to 2 to 3 was seen in re-operations for recurrence after which a metastasis was discovered. This could explain our higher finding of grade 1 and 2 tumors (78%) relative to the previous systematic review. Alternatively, the broader range of cases in our analysis could equally explain this difference among a relatively small number of cases.

While the CNS WHO grading system is the current gold standard, up to 20% of grade 1 meningiomas can develop aggressive features [37]. Many centers, including ours, favor examining the methylation status as an additional prognostic indicator. Grade 1 primary tumors made up the greatest proportion in the data. As the TTM in many cases was in the order of decades, it is possible that the primary tumor and its recurrence evolved into more malignant grade 2 or 3 histology before metastasizing. As such, we cannot conclude that the initial grade after primary resection impacts the TTM directly. We did find a statistically significant difference in the TTM in grade 1 and 2 primaries compared to grade 3. Moreover, due to the static nature of imaging and biopsy results, we cannot know when, or if, the malignant change and metastatic spread occurs.

### The need for improved diagnostic power

Central to calculating the risk of progression to MM is to identify which primary meningiomas have this malignant potential. The CNS WHO grading has so far been the gold standard in assessing the spectrum of aggressiveness of meningiomas through histopathological grading [26]. As there are no specific guidelines for how to approach a patient in whom metastasis is suspected, beyond the fact that grade 2 and 3 tumors have an increased risk of doing so [16], the post-operative work-up, including imaging, will be key; in this way, the initial CNS WHO grade may have a lesser role in the future. The initial histopathology may suggest that the patient may be at a higher risk of a malignant meningioma, but clear steps on how to stratify these patients further are needed. Several tailored imaging modalities have been suggested. Nuclear medicine techniques such as ^68^Ga-DOTANOC PET/CT imaging have been used to detect extracranial metastases with high signal intensity [17], relying on the high expression of somatostatin receptors in the cell membranes of meningiomas, and by extension, its metastases. Similarly, somatostatin receptor scintigraphy tagged with the ^111^indium-octreotide [1] and ^111^In-pentreotide [25] radioisotopes has shown to be useful for identifying extracranial masses. While these imaging modalities have shown potential, it will be important to integrate them into a comprehensive screening process. Corniola et al. suggested the use of CT chest abdomen pelvis imaging on all patients with grade 3 meningiomas at biopsy as a high-yield way of picking up those with the highest risk of metastatic disease [9]. However, as we have seen, grade 1 and 2 meningiomas also metastasize, and not that infrequently. Alternatively, Dalle Ore et al. have employed a screening paradigm including the somatostatin-targeting DOTATATE PET in which their number needed to screen for one biopsy-confirmed diagnosis of metastatic disease was 3.83 [10].

### A failure of controlling primary disease

Screening of all meningioma patients for this rare manifestation with the tools available to us today is likely not feasible; at the root of the problem is the inability to control primary disease. We summarized the novel treatments trialed between the years 2002 and 2022 (Table 2); none of the treatments prevented the progression of disease. While some cases in the literature were reported to have no further recurrence in primary or metastatic disease after resection of the metastatic lesion [11, 23, 31], these are rare reports and it is not clear what their patients’ course was after publication. Very few reports mention survival data, and additionally, if the patients were still alive at the time of publishing, then naturally it is not possible to interpret further (Supplementary Table 3). From this descriptive analysis, we found that treatment options and druggable targets for MM are lacking. Indeed, in our case series we carried out extensive cancer panel analyses with genomic screening in patients where it was feasible to do so and found no druggable targets. We also looked to enroll our patients into clinical studies; no such trials were available in the UK. There is a clearly a need for these trials in a condition we believe is under reported in the literature.

### Genomic and epigenomic approaches

Genomic and epigenomic analyses have enabled the understanding of meningiomas on a finer level than can be achieved with histopathology alone; these approaches could be the key to improved diagnosis and, crucially, druggable target discovery. These relatively novel efforts include mutation analysis and the generation of new classifications based on location and severity [7, 14, 44, 45], chromosomal instability [3, 19, 27], as well as familial syndromes associated with meningiomas [24]. More recently, epigenomic markers are giving rise to novel integrated classifications [2, 6, 28, 32, 38]. In the context of metastatic meningiomas, data on this remains sparse. However, recent exome sequencing on a patient with multiple metastases throughout the body has identified over 30 mutations that were found in both the primary and metastatic biopsies [20]. Interestingly, the primary tumor had almost 400 mutations that were not detected in the metastatic lesions. They also identified mutations that were not previously documented in aggressive meningiomas, including *CASS4* and *CMKLR1*, and the less frequently reported *MUC3A*, *ALDH1A3*, and *HOXA1*. In a similar way, stratifying patients by a combined genome-epigenome status could be a feasible alternative in future screening for MM.

### Global distribution and burden

Most reported cases of metastasis are from high-income countries; while this is not surprising, it does highlight that MM may be an underestimated problem from a global perspective. Therefore, any screening proposal for MM in the future should be sufficiently practical and feasible to be expanded globally. Novel genomic and epigenomic approaches are, at present, expensive and not readily accessible diagnostic procedures. However, an appreciation of the other observations described here, including the most common primary and metastatic sites in MM, could contribute to a novel diagnostic work-up approach.

### Limitations

The limitations of our study are partly due to the relative paucity of available literature on this topic. Since 1979 there have been a relatively low number of cases described, and not all report key information. Due to the relatively rare nature of this phenomenon, it is highly likely that the available literature does not reflect the global burden of this condition—a portion of the morbidity and mortality in meningioma patients could therefore be down to undetected metastases. There is also a bias in the reports used in this study to those from high- and upper-middle income countries. The burden in lower-middle and low-income countries could be much higher [34]. There have also been changes to WHO grading over time; no cases prior to 1979 detail a CNS WHO grade. Furthermore, the cases from earlier on in the twentieth century use terms to describe the histology that are now distinct from meningiomas—such as sarcomas or hemangiopericytomas. Lastly, there are controversies regarding some of the criteria in the CNS WHO classifications, such as the use of brain invasion as a stand-alone diagnostic criterion for grade 2 meningiomas [26, 35].

## Conclusion

In this study we have highlighted the unique clinical challenges in managing patients with metastatic meningioma. We reported that the convexity was the most likely intracranial primary location, that the initial CNS WHO grade at first available biopsy significantly affects the TTM, and that the lung is the most common site of metastasis. There is a lack of consensus on how to effectively manage these patients and invariably the outcomes are very poor. This dire situation is also seen in the management of recurrent intracranial WHO grade 2 and 3 meningiomas and reflects the paucity of drug therapies for these tumors. While metastatic meningiomas represent an aggressive and extreme end of the spectrum, the failure to manage them also reflects on the failure to control the primary disease. Only with advances in this area will we make progress with metastatic meningiomas. This approach will also be central to the care of these patients in poorly resourced communities around the world. The outcomes for metastatic meningioma are poor even in centers treating large numbers of meningioma patients, such as ours; there is an urgent need for multi-center clinical trials for this underestimated challenge. Only then may we hope to leverage our expanding knowledge on this rare but aggressive subset of meningiomas.

### Supplementary Information

Below is the link to the electronic supplementary material.

Supplementary file1 (DOCX 103 KB)

## Data Availability

Source data is provided in the supplementary material accompanying this paper. Any other data or information needed to re-produce the findings presented are available from the corresponding author upon reasonable request whilst also patient confidentiality.
