# Supplementary material for: Metastatic meningioma: a case series and systematic review
Source: Acta Neurochir (Wien). 2023 Jul 26;165(10):2873–83. doi: 10.1007/s00701-023-05687-3 (PMC10542723; doi:10.1007/s00701-023-05687-3)

**Supplementary Materials**

*Contains 3 supplementary tables and 1 supplementary figure, as referenced in manuscript.*

**Supp. Table 1: Summary of studies of MM in our systematic review of the literature – studies from 1979-2022.** NR – not reported; HIC – high-income country; LMIC – low- and middle-income country; TTM – time to metastasis.

| no | Author | Year | Country | Category | Age | Sex | Primary location | Grade | TTM | Metastasis location(s) |
| --- | --- | --- | --- | --- | --- | --- | --- | --- | --- | --- |
| 155 | Himic et al | 2022 | UK | HIC | 59 | F | Convexity | 3 | 7 | Liver, Soft tissue |
| 154 | Himic et al | 2022 | UK | HIC | 70 | F | Convexity | 2 | 14 | Soft tissue |
| 153 | Himic et al | 2022 | UK | HIC | 63 | M | Parasagittal | 3 | 1 | Lung |
| 152 | Himic et al | 2022 | UK | HIC | 47 | M | Convexity | 3 | 2 | Bone |
| 151 | Himic et al | 2022 | UK | HIC | 46 | M | Base | 2 | 3 | Lung, Other, Bone |
| 150 | Utsumi et al. | 2022 | JP | HIC | 75 | M | Convexity | 2 | 2 | Lung |
| 149 | Chew et al. | 2021 | MY | LMIC | 68 | F | Convexity | 1 | 16 | Lymph |
| 148 | Sarwan et al. | 2021 | US | HIC | 57 | M | Convexity | 1 | 9 | Liver |
| 147 | Mardani et al. | 2021 | IR | LMIC | 37 | F | Convexity | 1 | NR | Lung |
| 146 | Matsumura et al. | 2021 | JP | HIC | 75 | M | NR | 1 | 29 | Soft tissue, Bone |
| 145 | Schwock et al. | 2021 | CA | HIC | 47 | F | Convexity | 1 | 19 | Soft tissue |
| 144 | Nguyen et al. | 2021 | VN | LMIC | 40 | F | Convexity | 2 | 15 | Soft tissue |
| 143 | Sheng et al. | 2021 | CN | LMIC | 52 | F | Convexity | 1 | 10 | Liver |
| 142 | Ronen et al. | 2020 | US | HIC | 51 | M | Convexity | 2 | 19 | Other |
| 141 | He et al. | 2020 | CN | LMIC | 39 | M | Convexity | 3 | 7 | Lung, Soft tissue |
| 140 | Dincer et al. | 2020 | US | HIC | 41 | M | Parasagittal | 1 | 13 | Lung |
| 139 | Limarzi et al. | 2020 | IT | HIC | 68 | M | NR | 2 | 6 | Liver |
| 138 | Shimowaka et al. | 2020 | JP | HIC | 58 | F | Convexity | 1 | 9 | Liver |
| 137 | Enomoto et al. | 2019 | JP | HIC | 65 | M | Convexity | 1 | 26 | Liver |
| 136 | Dalle Ore et al. | 2019 | US | HIC | 69 | F | Convexity | 3 | 5.4 | Lung, Liver |
| 135 | Dalle Ore et al. | 2019 | US | HIC | 75 | F | Falx | 2 | 9.2 | Lung |
| 134 | Dalle Ore et al. | 2019 | US | HIC | 54 | F | Falx | 3 | 4.7 | Liver |
| 133 | Dalle Ore et al. | 2019 | US | HIC | 64 | F | Parasagittal | 3 | 10.8 | Soft tissue |
| 132 | Dalle Ore et al. | 2019 | US | HIC | 82 | F | Convexity | 2 | 26.2 | Bone |
| 131 | Dalle Ore et al. | 2019 | US | HIC | 53 | F | Falx | 2 | 26.2 | Liver |
| 130 | Dalle Ore et al. | 2019 | US | HIC | 50 | M | Base | 2 | 27.6 | Lung, Liver |
| 129 | Dalle Ore et al. | 2019 | US | HIC | 69 | M | Base | 2 | 31 | Liver |
| 128 | Vakil et al. | 2019 | US | HIC | 91 | F | Convexity | 3 | 5 | Lung |
| 127 | Woo et al. | 2019 | CN | LMIC | 37 | F | Convexity | 1 | 11 | Lung |
| 126 | Sathirareuangchai et al. | 2019 | US | HIC | 59 | F | Convexity | 1 | NR | Lung |
| 125 | Soukup et al. | 2019 | CZ | HIC | 54 | M | Convexity | 3 | 1.5 | Other |
| 124 | Rajadurai et al. | 2019 | NZ | HIC | 52 | M | Falx | 2 | 13 | Lung |
| 123 | Baeesa et al. | 2018 | SA | HIC | 29 | M | Convexity | 1 | 8 | Bone |
| 122 | Simonetti et al. | 2018 | IT | HIC | 25 | F | Parasagittal | 1 | 24 | Lung, Other |
| 121 | Kobayashi et al. | 2018 | JP | HIC | 64 | M | Parasagittal | 3 | 2 | Soft tissue |
| 120 | Hu et al. | 2018 | CN | LMIC | 58 | M | Convexity | NR | NR | Lung |
| 119 | Corniola et al. | 2017 | CH | HIC | 74 | F | Base | 3 | 2 | Lung |
| 118 | Thomas and Dalal | 2017 | US | HIC | 58 | F | Convexity | 3 | 2 | Lung, Soft tissue |
| 117 | Kessler et al. | 2017 | US | HIC | 24 | F | Falx | 1 | 9 | Lung |
| 116 | Kessler et al. | 2017 | US | HIC | 69 | F | Falx | 3 | 3 | Lung |
| 115 | Kessler et al. | 2017 | US | HIC | 54 | F | Convexity | 2 | 28 | Lung, Soft tissue |
| 114 | Kessler et al. | 2017 | US | HIC | 66 | M | Convexity | 3 | 3 | Lung, Soft tissue |
| 113 | Kessler et al. | 2017 | US | HIC | 65 | M | Convexity | 2 | 6 | Liver, Bone |
| 112 | Kessler et al. | 2017 | US | HIC | 49 | M | Falx | 2 | 7 | Liver |
| 111 | Zhao et al. | 2017 | CN | LMIC | 39 | F | Convexity | 3 | 0.04 | Lung, Liver, Other, Soft tissue |
| 110 | Cho and Yoon | 2017 | KR | HIC | 61 | F | Convexity | 1 | 12 | Lung |
| 109 | Paix et al. | 2017 | FR | HIC | 43 | F | Convexity | 2 | NR | Bone, Lung, Liver |
| 108 | McCarthy et al. | 2016 | UK | HIC | 59 | M | Falx | 2 | 2 | Soft tissue, Liver, Bone |
| 107 | Singh et al. | 2016 | US | HIC | 58 | F | Base | 2 | 3 | Bone |
| 106 | Singh et al. | 2016 | US | HIC | 57 | F | Convexity | 1 | 6 | Bone |
| 105 | Wang et al. | 2016 | CN | LMIC | 56 | M | Convexity | 2 | 3 | Lung |
| 104 | Chua et al. | 2016 | SG | HIC | 26 | F | Parasagittal | 2 | 7 | Bone, Liver, Soft tissue |
| 103 | Kakkar et al. | 2016 | IN | LMIC | 31 | M | Falx | 3 | 13 | Lung |
| 102 | Leemans et al. | 2016 | BE | HIC | 62 | M | Falx | 1 | 22 | Lung |
| 101 | Leemans et al. | 2016 | BE | HIC | 68 | M | Falx | 3 | 15 | Lung, Soft tissue |
| 100 | Parameshwaran Nair et al. | 2015 | IN | LMIC | 59 | M | Convexity | 3 | 7 | Other |
| 99 | Wang et al. | 2015 | CN | LMIC | 54 | F | Base | 1 | 2.5 | Lung |
| 98 | Strong et al. | 2015 | US | HIC | 57 | F | Convexity | 1 | 25 | Bone |
| 97 | Golemi et al. | 2015 | IT | HIC | 65 | M | Falx | 1 | 3 | Lung, Soft tissue, Lymph |
| 96 | Frydrychowicz et al. | 2015 | DE | HIC | 72 | F | Convexity | 2 | 11 | Lung |
| 95 | Frydrychowicz et al. | 2015 | DE | HIC | 45 | F | Convexity | 2 | 5 | Lung |
| 94 | Kawaji et al. | 2015 | JP | HIC | 70 | F | Base | 2 | 2 | Bone, Liver, Soft tissue |
| 93 | Ocque et al. | 2014 | US | HIC | 71 | F | Convexity | NR | NR | Liver |
| 92 | Ocque et al. | 2014 | US | HIC | 47 | F | NR | NR | NR | Lung |
| 91 | Ocque et al. | 2014 | US | HIC | 43 | F | NR | NR | NR | Bone, Lung, Liver |
| 90 | Forest et al. | 2014 | FR | HIC | 80 | F | Convexity | 1 | 15 | Liver, Bone |
| 89 | Forest et al. | 2014 | FR | HIC | 68 | M | Base | 1 | 12 | Lung |
| 88 | Forest et al. | 2014 | FR | HIC | 68 | M | Base | 1 | 2 | Liver, Bone |
| 87 | Tao et al. | 2014 | CN | LMIC | 51 | F | Other | 3 | 1 | Lung, Bone |
| 86 | Nakayama et al. | 2014 | JP | HIC | 25 | F | Convexity | 1 |  | Lung, Soft tissue |
| 85 | Gupta et al. | 2013 | US | HIC | 72 | M | Parasagittal | 3 | 4 | Bone |
| 84 | Lanfranchi and Nikpoor | 2013 | US | HIC | 74 | M | Base | 2 | 4 | Lung, Liver |
| 83 | Moubayed et al. | 2013 | US | HIC | 58 | M | Convexity | 2 | 4 | Lymph |
| 82 | Nakano et al. | 2012 | JP | HIC | 34 | M | Parasagittal | 1 | 4 | Lung |
| 81 | Celenk et al. | 2012 | TR | LMIC | 60 | F | Base | 1 | 19 | Soft tissue |
| 80 | Kim et al. | 2012 | KR | HIC | 74 | F | Convexity | 3 | 5 | Bone |
| 79 | Kanzaki et al. | 2011 | JP | HIC | 67 | F | Convexity | 2 | 15 | Lung |
| 78 | Alexandru et al. | 2011 | US | HIC | 67 | M | Convexity | 3 | NR | Lung |
| 77 | Alexandru et al. | 2011 | US | HIC | 26 | F | Convexity | 3 | NR | Lung |
| 76 | Alexandru et al. | 2011 | US | HIC | 84 | F | Convexity | 1 | NR | Lung |
| 75 | Alexandru et al. | 2011 | US | HIC | 38 | M | Base | 2 | NR | Lung |
| 74 | Alexandru et al. | 2011 | US | HIC | 52 | F | Convexity | 2 | NR | Lung |
| 73 | Alexandru et al. | 2011 | US | HIC | 57 | M | Convexity | 3 | NR | Lung |
| 72 | Lambertz et al. | 2011 | DE | HIC | 65 | F | Convexity | 2 | 12 | Lung, Liver, Soft tissue |
| 71 | Sabet et al. | 2011 | DE | HIC | 62 | F | Convexity | 3 | NR | Lung |
| 70 | Taieb et al. | 2011 | FR | HIC | 30 | F | Base | 1 | 30 | Lung, Liver, Soft tissue |
| 69 | Brennan et al. | 2010 | IE | HIC | 74 | F | Parasagittal | 2 | 22 | Lung |
| 68 | Psaras et al. | 2009 | DE | HIC | 65 | F | Convexity | 1 | 15 | Lung |
| 67 | Estanislau et al. | 2009 | BR | LMIC | 75 | M | Convexity | 2 | 6 | Lung, Bone |
| 66 | Lee et al. | 2009 | KR | HIC | 68 | M | Other | 2 | 1.2 | Bone, Lung, Soft tissue |
| 65 | Sujit Kumar et al. | 2009 | IN | LMIC | 46 | F | Convexity | 1 | 3 | Bone |
| 64 | Fulkerson et al. | 2008 | US | HIC | 54 | M | Other | 1 | NR | Lung |
| 63 | Azene et al. | 2008 | US | HIC | 78 | F | Convexity | 1 | 9 | Bone, Soft tissue |
| 62 | Gladin et al. | 2007 | IT | HIC | 58 | M | Base | 1 | 13 | Lung |
| 61 | Gladin et al. | 2007 | IT | HIC | 47 | M | Convexity | 1 | 9 | Lung |
| 60 | Gladin et al. | 2007 | IT | HIC | 40 | M | Convexity | 1 | 7 | Bone |
| 59 | Asioli et al. | 2007 | IT | HIC | 58 | F | NR | 1 | 12 | Lung |
| 58 | Chuang et al. | 2006 | CN | LMIC | 52 | M | Convexity | 2 | 0.3 | Bone |
| 57 | Vik et al. | 2006 | NO | HIC | 54 | M | Parasagittal | 2 | 9.5 | Lung, Soft tissue |
| 56 | Fabi et al. | 2006 | IT | HIC | 57 | F | Convexity | 3 | 1 | Lung, Bone |
| 55 | Delgado-Lopez et al. | 2006 | ES | HIC | 37 | M | Other | 1 | 8 | Bone |
| 54 | Teague and Conces | 2005 | US | HIC | 64 | M | Convexity | 2 | 8 | Lung |
| 53 | D'Aiuto et al. | 2005 | IT | HIC | 71 | M | Convexity | 1 | 7 | Lung |
| 52 | Erman et al. | 2005 | TR | LMIC | 34 | F | Parasagittal | 1 | 8 | Lung |
| 51 | Knoop et al. | 2004 | DE | HIC | 53 | M | Convexity | 1 | NR | Lung |
| 50 | Pistolesi et al. | 2004 | IT | HIC | 34 | M | Convexity | 2 | 1 | Lung |
| 49 | Dogan et al. | 2004 | TR | LMIC | 27 | M | Convexity | NR | 0.8 | Lung, Bone |
| 48 | Travitzky et al. | 2003 | IL | HIC | 60 | F | NR | 3 | 19 | Lung |
| 47 | Cerd -Nicol s et al. | 2003 | ES | HIC | 75 | M | Parasagittal | 1 | 0.3 | Lung, Liver |
| 46 | Pramesh et al. | 2003 | IN | LMIC | 29 | F | Convexity | 1 | 9 | Lung, Soft tissue |
| 45 | Kovoor et al. | 2002 | IN | LMIC | 40 | F | Convexity | 1 | 2 | Lung |
| 44 | Lee et al. | 2002 | TW | LMIC | 48 | F | Convexity | 1 | 3 | Bone |
| 43 | Fuentes et al. | 2002 | FR | HIC | 63 | F | Convexity | 2 | 10 | Bone |
| 42 | Kaminski et al. | 2001 | US | HIC | 68 | M | Base | 3 | 3 | Lung |
| 41 | Williamson et al. | 2001 | US | HIC | 64 | F | Convexity | NR | 13 | Soft tissue, Bone |
| 40 | Kros et al. | 2000 | NL | HIC | 13 | M | Base | 3 | 1 | Soft tissue |
| 39 | Drummond et al. | 2000 | AU | HIC | 76 | M | Convexity | 2 | 10 | Lung |
| 38 | Baisden et al. | 1999 | US | HIC | 71 | F | Base | I2 | 13 | Lung |
| 37 | Figueroa et al. | 1999 | US | HIC | 50 | F | Base | 1 | 5 | Lung, Liver |
| 36 | Adlakha et al. | 1999 | US | HIC | 30 | M | Parasagittal | 3 | 6 | Lung |
| 35 | Adlakha et al. | 1999 | US | HIC | 70 | F | Parasagittal | 1 | NR | Lung |
| 34 | Adlakha et al. | 1999 | US | HIC | 17 | F | Convexity | 3 | 6 | Lung |
| 33 | Sironi et al. | 1997 | IT | HIC | 46 | M | Convexity | 1 | 12 | Lung |
| 32 | Shin et al. | 1996 | US | HIC | 53 | F | Convexity | 1 | 9 | Lung |
| 31 | Enam et al. | 1996 | US | HIC | 73 | F | Convexity | NR | 1 | Bone, Liver |
| 30 | Enam et al. | 1996 | US | HIC | 52 | F | Base | NR | 1.4 | Bone |
| 29 | Hishima et al. | 1995 | JP | HIC | 25 | F | Convexity | 1 | NR | Lung |
| 28 | Palmer et al. | 1994 | UK | HIC | 55 | M | Base | NR | 12 | Lung |
| 27 | Kodama et al. | 1991 | JP | HIC | 61 | F | Convexity | 1 | 19 | Lung, Soft tissue |
| 26 | Tao | 1991 | CN | LMIC | 32 | F | Convexity | NR | 18 | Lung |
| 25 | Latz et al. | 1991 | DE | HIC | 29 | NR | Falx | NR | 2 | Bone |
| 24 | Leighton et al. | 1991 | US | HIC | 55 | M | Convexity | 1 | 9 | Soft tissue |
| 23 | Ng et al. | 1990 | CN | HIC | 66 | M | Parasagittal | 1 | NR | Lung |
| 22 | Clavere et al. | 1990 | FR | HIC | 41 | F | Parasagittal | 1 | 18 | Bone |
| 21 | Fukushima et al. | 1989 | JP | HIC | 40 | M | Base | 3 | 8 | Lung |
| 20 | Slavin | 1989 | US | HIC | 71 | F | Base | 2 | 2 | Lung |
| 19 | Lemay et al. | 1989 | US | HIC | 56 | F | Base | 1 | 10 | Lung |
| 18 | Som et al. | 1987 | US | HIC | 53 | F | Base | NR | 5 | Bone |
| 17 | Som et al. | 1987 | US | HIC | 50 | M | Parasagittal | NR | 5 | Lung |
| 16 | Som et al. | 1987 | US | HIC | 56 | M | Convexity | NR | 7 | Soft tissue |
| 15 | Stoller et al. | 1987 | US | HIC | 63 | F | Convexity | NR | 18 | Lung |
| 14 | Jenkinson et al. | 1987 | UK | HIC | 38 | M | Convexity | NR | 5 | Liver, Soft tissue |
| 13 | Noterman et al. | 1987 | BG | HIC | 35 | M | Base | NR | 1 | Bone |
| 12 | Aumann et al. | 1986 | NL | HIC | 45 | F | Parasagittal | 1 | 5 | Lung |
| 11 | Miller et al. | 1985 | US | HIC | 61 | M | Convexity | 1 | NR | Lung |
| 10 | Ishikura et al. | 1983 | JP | HIC | 53 | M | NR | NR | 4 | Bone |
| 9 | Ishibashi et al. | 1983 | JP | HIC | 21 | F | Convexity | NR | 2 | Lung, Liver |
| 8 | Barz et al. | 1982 | DE | HIC | 59 | F | Base | 2 | NR | Other |
| 7 | Salvati | 1981 | US | HIC | 29 | M | Convexity | NR | 6 | Bone |
| 6 | Pasquier et al. | 1986 | FR | HIC | 29 | M | Parasagittal | NR | 6 | Lung |
| 5 | Inoue et al. | 1983 | JP | HIC | 56 | F | Parasagittal | NR | 7 | Lung, Liver |
| 4 | Wende et al. | 1983 | DE | HIC | 35 | M | NR | NR | 2 | Lung |
| 3 | Jennings and Cook | 1983 | UK | HIC | 37 | M | Base | NR | 4 | Bone |
| 2 | Thomas et al. | 1981 | CN | LMIC | 33 | M | Convexity | NR | 1 | Other |
| 1 | Thomas et al. | 1981 | CN | LMIC | 42 | M | Convexity | NR | 6 | Lung |

**Supplementary Table 2: Summary of the frequency of the histological subtypes reported in the biopsy of the primary meningiomas.** Note that not all of the reports reference the exact histological subtype, sometimes only the CNS WHO grade with a verbal description but no conclusive histological definition – these have not been included in this table.

| **CNS WHO Grade 1** | **Reported** |
| --- | --- |
| Meningothelial | 19 |
| Transitional | 14 |
| Fibrous | 7 |
| Psammomatous | 3 |
| Angiomatous | 1 |
| **CNS WHO Grade 2** |  |
| Anaplastic | 12 |
| **CNS WHO Grade 3** |  |
| Atypical | 28 |
| Rhabdoid | 5 |
| Papillary | 4 |

**Supplementary Table 3: Available survival data for metastatic meningiomas.** From those studies that reported the survival information for cases, they have been included here. Most studies did not report this metric. Reported from the time of initial diagnosis.

| **Author** | **Year** | **Survival from**  **first diagnosis (years)** |
| --- | --- | --- |
| Matsumura et al. | 2021 | 33 |
| Schwock et al. | 2021 | 24 |
| Dalle Ore et al. | 2019 | 24 |
|  |  | 39 |
| Baeesa et al. | 2018 | 6 |
| Simonetti et al. | 2018 | 24 |
| Corniola et al. | 2017 | 2 |
| Thomas and Dalal | 2017 | 4 |
| Kessler et al. | 2017 | 15 |
|  |  | 5 |
|  |  | 32 |
|  |  | 3 |
|  |  | 10 |
|  |  | 7 |
| Zhao et al. | 2017 | 1 |
| Singh et al. | 2016 | 3.75 |
|  |  | 7 |
| Chua et al. | 2016 | 7 |
| Kakkar et al. | 2016 | 14 |
| Leemans et al. | 2016 | 18 |
| Kawaji et al. | 2015 | 2.6 |
| Forest et al. | 2014 | 4 |
| Tao et al. | 2014 | 1.7 |
| Lambertz et al. | 2011 | 1.6 |
| Taieb et al. | 2011 | 31 |
| Estanislau et al. | 2009 | 8 |
| Lee et al. | 2009 | 2 |
| Erman et al. | 2005 | 8 |
| Dogan et al. | 2004 | 1.5 |
| Kros et al. | 2000 | 2 |
| Adlakha et al. | 1999 | 10 |
| Enam et al. | 1996 | 2 |
| Palmer et al. | 1994 | 13 |
| Clavere et al. | 1990 | 18 |
| Slavin | 1989 | 2 |
| Lemay et al. | 1989 | 18 |
| Som et al. | 1987 | 5 |
|  |  | 5.5 |
| Noterman et al. | 1987 | 5 |
| Ishibashi et al. | 1983 | 3 |
| Barz et al. | 1982 | 11 |

**Supp. Fig.1: No relationship between the age of the patient and time to first extracranial metastasis.** Linear regression reveals no correlation between age and time to metastases (*R squared* = 0.02, *p* = 0.17, *Spearman’s Rank Correlation test*). Shaded area illustrates SEM.


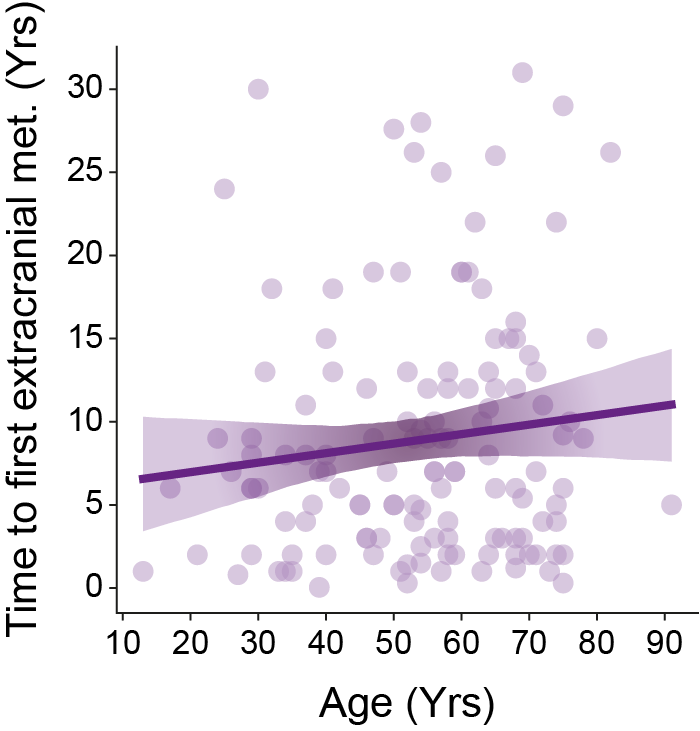

Supplement: Supplementary file 1 — Supplementary file1 (DOCX 103 KB) [file 701_2023_5687_MOESM1_ESM.docx]
